# Utilization of Phenol as Carbon Source by the Thermoacidophilic Archaeon *Saccharolobus solfataricus* P2 Is Limited by Oxygen Supply and the Cellular Stress Response

**DOI:** 10.3389/fmicb.2020.587032

**Published:** 2021-01-08

**Authors:** Jacqueline Wolf, Julia Koblitz, Andreas Albersmeier, Jörn Kalinowski, Bettina Siebers, Dietmar Schomburg, Meina Neumann-Schaal

**Affiliations:** ^1^Department of Bioinformatics and Biochemistry, Technische Universität Braunschweig, Braunschweig, Germany; ^2^Braunschweig Integrated Centre of Systems Biology (BRICS), Braunschweig, Germany; ^3^Center for Biotechnology-CeBiTec, Universität Bielefeld, Bielefeld, Germany; ^4^Molecular Enzyme Technology and Biochemistry (MEB), Environmental Microbiology and Biotechnology (EMB), Centre for Water and Environmental Research (CWE), University of Duisburg-Essen, Essen, Germany; ^5^Junior Research Group Bacterial Metabolomics, Leibniz Institute DSMZ German Collection of Microorganisms and Cell Cultures, Braunschweig, Germany

**Keywords:** phenol biodegradation, *Sulfolobales*, stress response, metabolic modeling, systems biology

## Abstract

Present in many industrial effluents and as common degradation product of organic matter, phenol is a widespread compound which may cause serious environmental problems, due to its toxicity to animals and humans. Degradation of phenol from the environment by mesophilic bacteria has been studied extensively over the past decades, but only little is known about phenol biodegradation at high temperatures or low pH. In this work we studied phenol degradation in the thermoacidophilic archaeon *Saccharolobus solfataricus* P2 (basonym: *Sulfolobus solfataricus*) under extreme conditions (80°C, pH 3.5). We combined metabolomics and transcriptomics together with metabolic modeling to elucidate the organism’s response to growth with phenol as sole carbon source. Although *S. solfataricus* is able to utilize phenol for biomass production, the carbon source induces profound stress reactions, including genome rearrangement as well as a strong intracellular accumulation of polyamines. Furthermore, computational modeling revealed a 40% higher oxygen demand for substrate oxidation, compared to growth on glucose. However, only 16.5% of oxygen is used for oxidation of phenol to catechol, resulting in a less efficient integration of carbon into the biomass. Finally, our data underlines the importance of the phenol *meta*-degradation pathway in *S. solfataricus* and enables us to predict enzyme candidates involved in the degradation processes downstream of 2-hydroxymucconic acid.

## Introduction

Phenol is a widespread environmental pollutant, present in the effluents of various industries including coal conversion, production of phenolic resins and the textile industry (Naguib and Badawy, 2020). If it is not removed from ground water, phenol could cause serious health problems to humans, animals and aquatic life (Saha et al., 1999; Michalowicz and Duda, 2007). Additionally, phenolic compounds can result from degradation of natural organic matter in the environment, e.g., from the decomposition of plants and animals (Anku et al., 2017), and are thus ubiquitous in the environment. A number of mesophilic bacteria and yeasts can degrade phenol, e.g., *Acinetobacter radioresistens*, *Glutamicibacter nicotianae*, and *Candida subhashii* (Filipowicz et al., 2017; Duraisamy et al., 2020; Liu et al., 2020). The mechanism of phenol degradation in mesophilic microorganisms has been thoroughly studied because of their application in bioremediation. Although there have been several studies on thermophilic phenol degradation in *Bacilli* (Gurujeyalakshmi and Oriel, 1989; Antranikian, 1998; Duffner et al., 2000), *Saccharolobus solfataricus* (basonym: *Sulfolobus solfataricus*) was the first hyperthermophilic archaeon reported to be able to grow aerobically on phenol (Izzo et al., 2005). Since then this organism has been studied extensively with respect to its phenol degrading capacity and evidence has been found for an active phenol *meta*-degradation pathway in *S. solfataricus* (Christen et al., 2011, 2012; Comte et al., 2013). However, only little is known on the participating enzymes in the degradation process, as the homology of archaeal proteins to their bacterial counterparts is often very low. Only the monooxygenase gene cluster and the catechol-2,3-diooxygenase of *S. solfataricus* have been characterized so far (Izzo et al., 2005; Chae et al., 2007).

In this work we combined data from transcriptome and metabolome analysis together with metabolic modeling, to explore the global changes in *S. solfataricus* P2 in response to growth on phenol as sole carbon source. Our data strengthen the evidence for the activity of the *meta-*degradation route and predict gene candidates encoding for the missing proteins of this pathway in this hyperthermophilic archaeon.

## Materials and Methods

### Strain and Growth Conditions

*Saccharolobus solfataricus* P2 (Zillig et al., 1980; Sakai and Kurosawa, 2018) was cultivated in long neck flasks at 80°C, pH 3.5 and 160 rpm (Thermotron, Infors AG, Switzerland) on defined minimal medium (Brock et al., 1972), containing either 10 mM phenol or 22.2 mM D-glucose as sole carbon source. For adaption to phenol, pre-cultures, containing 2 mM D-glucose and 5 mM phenol, were inoculated with glucose adapted stock cultures, prepared as described earlier (Zaparty et al., 2010). These pre-cultures were used to inoculate the main cultures to an OD_600_ of ∼0.05. D-glucose main cultures were directly inoculated, using glucose adapted stock cultures. Cell growth was monitored photometrically, following the increase in optical density at 600 nm. To monitor phenol consumption, 1 ml samples were taken in regular intervals, cells were harvested by centrifugation (20,000 g; 5 min, 20°C) and supernatants were stored at −20°C until further processing. Determination of major biomass fractions was performed as described previously (Wolf et al., 2016). All phenotypic data for growth on D-glucose (growth rate, substrate uptake rate, and biomass composition) have been published previously (Wolf et al., 2016).

### Extraction of Intra- and Extracellular Metabolites and Coenzyme A Derivatives

Phenol consumption was measured by GC-MS analysis, following the protocol for extraction, measurement and data processing as described previously (Neumann-Schaal et al., 2015). Briefly, 300 μl phenol-containing supernatant was mixed with 200 μl o-xylene as internal standard. After addition of 20 μl H_2_SO_4_ (49–51%), 200 μl methyl-*tert-*butylether was used to extract phenol. The extract was directly injected into the GC-MS without further derivatization. For calculation of absolute concentrations, an external phenol calibration curve ranging from 2 to 10 mM was measured.

Intracellular metabolites, coenzyme A derivatives and supernatants for analysis of secreted compounds were extracted, measured and analyzed as described earlier (Zaparty et al., 2010; Wolf et al., 2016). Briefly, for intracellular metabolome analysis, cells corresponding to 15 mg cell dry weight were harvested and washed twice with 0.9% NaCl. Afterward 1.5 ml methanol containing 30 μl ribitol (c = 0.2 g⋅l^–1^) as internal standard were added and cells were lysed in an ultrasonic bath at 70°C for 15 min. Subsequently, cells were chilled on ice and mixed with 1.5 ml deionized water. For extraction of metabolites, 1 ml of chloroform were added and samples were mixed vigorously. After phase separation the upper polar phase was collected and dried in a vacuum concentrator at 15°C overnight.

For extracellular metabolome analysis, supernatants were mixed with 20 μl ribitol (c = 0.2 g⋅l^–1^) as internal standard and dried in a vacuum concentrator overnight.

For extraction of Coenzyme A (CoA) derivatives, cells corresponding to 10 mg cell dry weight were harvested and resuspended in 1 ml methanol, containing 0.2 mg⋅l^–1^ biochanin A as internal standard. Cells were lysed using a Precellys 24 homogeniser (Peqlab, Germany) at −10°C with three cycles of homogenization (6,800 rpm, 30 s, with equivalent breaks). Afterward, the lysate was transferred to ice cold ammonium acetate solution (25 mM, pH 6) and cleared by centrifugation. CoA derivatives were extracted by solid phase extraction, using Strata XL-AW solid phase extraction columns (Phenomenex, Germany). Columns were sequentially equilibrated using 1 ml methanol, followed by 1 ml methanol:H_2_O:formic acid (50:45:5) and 1 ml H_2_O. After loading the cell lysate onto the column it was washed with 1 ml ammonium acetate (25 mM, pH 7.2) and dried at 700 mbar for 3 min. CoA derivatives were eluted using 1 ml methanol containing 5% (v/v) ammonia. Extracts were dried in a vacuum concentrator at 15°C overnight. For HPLC-MS analysis dried extracts were dissolved in 0.2 ml sample buffer (25 mM ammonium acetate pH 3.5, 2% (v/v) methanol).

### GC-MS Measurement of Intra- and Extracellular Metabolites and HPLC-MS Measurement of CoA Derivatives

Metabolites for intra- and extracellular metabolome analysis were derivatized, following the protocol described by Zaparty et al. (2010). Briefly, dried extracts were dissolved in 40 μl 20 mg⋅ml^–1^ methoxyamine hydrochloride in pyridine and incubated for 90 min at 30°C. Afterward 60 μl *N*-methyl-*N*-(trimethylsilyl)-trifluoro-acetamide was added and incubated for 30 min at 37°C, followed by 120 min at 25°C. After centrifugation 60 μl of derivatized extracts were transferred into glass vials for GC-MS analysis.

Metabolites were analyzed using a Leco Pegasus 4D GCxGC TOFMS (Leco, Germany), equipped with a 30 m, 0.25 mm Zebron ZB-5 ms column (Phenomenex, Germany). Ionization of analytes was performed by positive electron ionization (EI+). Gas chromatography was performed using a helium gas flow of 1.2 ml⋅min^–1^, using the following temperature gradient: 0.02 min at 70°C, increase to 330°C with 12°C⋅s^–1^, constant temperature period at 330°C for 5 min. Full scan mass spectra were recorded from 45 to 600 m/z.

Phenol consumption was monitored on a Thermo GC Ultra coupled to a DSQ II mass spectrometer Thermo Fisher Scientific, Schwerte, Germany). Chromatography was performed as described previously (Neumann-Schaal et al., 2015) using an Agilent VF-WAX-MS column (30 m, 0.25 mm diameter; Agilent, Santa Clara, United States) with the following temperature gradient: 1 min at 55°C, increase to 245°C with 10°C⋅min^–1^, constant temperature period at 245°C for 2 min. Full scan mass spectra were recorded from 20 to 400 m/z.

Analysis of CoA derivatives was performed on a Dionex ultimate 3,000 system (Thermo Fisher Scientific Inc., Schwerte, Germany) coupled to a Bruker MicroTOF QII mass spectrometer (Bruker Daltonik GmbH, Bremen Germany). Chromatography was performed based on the protocol described by Peyraud et al. (2009), using a C_18_ analytical column (Gemini 150 × 2.0 mm, particle size 3 μm; Phenomenex, Aschaffenburg, Germany). At a constant temperature of 35°C and a flow rate of 220 μl⋅min^–1^ the following gradient was used: 1 min 5% B (methanol): 95% A (50 mM ammonium formiate, pH 8.1); 18 min 30% B: 70% A; 7 min 95% B: 5% A; 4 min 95% B: 5% A. MS analysis was done in positive ESI mode with 3 Hz data acquisition and automated MS^2^ acquisition. Mass spectra were recorded from 90 to 1178 m/z.

### Data Processing and Statistical Analysis

Data analysis was performed as described previously (Wolf et al., 2016). Briefly, GC-MS data was processed using the MetaboliteDetector software for automated peak detection and deconvolution (Hiller et al., 2009; Zech et al., 2013). Compounds were identified by comparison of mass spectra and retention indices to a compound library which was created by merging our in-house library with the Golm metabolome database (Kopka et al., 2005). Peak areas were normalized to the internal standards and peak areas of different derivatives were summarized to the corresponding metabolites. Non-biological and artificial peaks were eliminated by the aid of blank samples. Finally, the data was normalized using a central normalization procedure to the reference D-glucose.

Internal mass calibration with the sodium formiate cluster and export of HPLC data to the mzXML format was performed using the Bruker DataAnalysis software (Bruker Daltonik GmbH, Bremen, Germany). Raw data was processed using the XCMS package for R (Smith et al., 2006; Benton et al., 2008; Tautenhahn et al., 2008). Peak identification was performed using the accurate masses of the [M+2H]^2+^ ions and retention times of synthetic CoA standards. If there was no synthetic standard available for a certain compound, both the calculation of the molecular mass from [M+2H]^2+^ and [M+H]^+^ ions and sum formula predictions using the accurate masses and the isotopic pattern were used for identification. MS^2^ fragmentation was used to confirm the presence of the CoA moiety. Peak areas were normalized by central normalization to the reference condition.

Statistical significant differences for all metabolites were determined by non-parametric Kruskal-Wallis test (Kruskal and Wallis, 1952), using the Benjamini–Yekutieli correction to control the false discovery rate (Benjamini and Yekutieli, 2001). Changes with a *p* < 0.01 were considered to be significant.

### RNA Isolation, Sequencing, and Data Analysis

RNA was extracted from frozen cell pellets using Trizol (Thermo Fisher Scientific, United States) as described earlier (Hottes et al., 2004). Afterward, ribosomal RNA was depleted using the *RiboZero magnetic kit* for bacteria (Epicenter, United States) according to the manufacturer’s protocol with slight modifications: only 90 μl of magnetic beads were used. For the rRNA removal reaction 1 μg was mixed with 4 μl removal solution in a total volume of 20 μl. Sequencing libraries for all samples and replicates were prepared with the TruSeq^®^ Stranded mRNA HT kit (Illumina) starting with the RNA fragmentation step after elution of precipitated RNA in 19 μl of the Fragment-Prime-Mix. Sequencing was performed on a HiSeq1500 instrument (Illumina) in rapid mode with a read length of 2 × 25 nt. Sequencing reads were mapped with Bowtie2 (Langmead and Salzberg, 2012) against the reference genome (*Saccharolobus solfataricus* P2, genome size: 2,992,245 nt, RefSeq ID: NC_002754.1) and calculation of mapped reads per gene were done using ReadXplorer (Hilker et al., 2014). Subsequently, RPKM values were computed for each gene (Mortazavi et al., 2008). To reduce influence of global and systemic changes on transcript abundance the data was additionally normalized by a central normalization procedure. To distinguish between background activity and active gene expression a mean log intensity (*A*-value, Dudoit et al., 2002) cut-off value of 2 was applied. Furthermore, only genes with an at least fourfold difference between both conditions and a RPKM value higher than the median RPKM value of the whole dataset (median RPKM = 69 ± 5.2) were included into the analysis. Statistical analysis was performed using DeSeq (Anders and Huber, 2010) and changes with a *p* < 0.01 were considered to be significant.

### Metabolic Modeling

The published metabolic model of *S. solfataricus* (Wolf et al., 2016; Stark et al., 2017) was used to elucidate changes in metabolic fluxes during growth on phenol. A glucose standard scenario was chosen as reference condition. Phenol degradation pathways were revised. Because of their importance in this study, the protons pumped by the two terminal oxidases SoxABCD and SoxM were also revised by the use of primary literature (Gleißner et al., 1997; Komorowski et al., 2002). The biomass composition for growth on phenol was added according to experimental data. The amount of polyamines in *S. solfataricus* grown on glucose was calculated from Giles and Graham (2008). The relative changes in metabolic polyamine levels were used to calculate the polyamine content for the growth on phenol. Transcriptomic and metabolomic measurements were included by limiting the metabolic fluxes within the model.

Metabolic modeling was performed using the metabolic modeling toolbox MMTB^1^. The metabolic model used during this study as well as glucose and phenol scenarios were uploaded to the MMTB platform to enhance further usage by the community. Data were visualized using MetaboMAPS (Helmecke et al., 2020). Swiss-Model was used to model the 3D-structure of proteins.

## Results

### Phenotypic Characterization of *S. solfataricus* P2 Growing on Phenol as Sole Carbon Source

Basic physiological parameters of *S. solfataricus* P2 were determined using Brock minimal medium (Brock et al., 1972) containing 10 mM phenol as sole carbon source. No growth was observed at phenol concentrations above 14 mM (Supplementary Material S1), indicating toxicity of the substrate at higher concentrations. Data for comparison with glucose as substrate (growth curve, substrate uptake rate and biomass composition) were published previously (Wolf et al., 2016). Overall, *S. solfataricus* showed a reduced growth rate and a lower biomass yield on 10 mM phenol compared to growth on 22 mM D-glucose (Supplementary Material S2).

Although the maximum substrate uptake rate of *S. solfataricus* P2 grown on phenol is comparable to growth on glucose, phenol has shown to be only a poor source of carbon and energy as a considerably decreased growth rate and less efficient integration of carbon into the biomass was observed (Table 1).

**TABLE 1:** Comparison of *in vivo* and *in silico* growth parameters.

	Phenol	D-glucose
μ*_*in vivo*_* [h^–1^]	0.015 ± 9.74⋅10**^–^**^5^	0.045 ± 0.001
μ*_*in silico*_* [h^–1^]	0.021 ± 0.068	0.063 ± 0.081
qS_*max*_ [mmol⋅g^–1^⋅h^–1^]	1.24 ± 0.20	1.13 ± 0.11
CDW_*max*_ [g⋅l^–1^]	0.26 ± 3.74⋅10**^–^**^3^	0.70 ± 0.01
Y_*C*_*_*in vivo*_* [%]	13.8 ± 3.1	46.5 ± 1.8
Y_*C*_*_*in silico*_* [%]	14.3 ± 2.2	43.5 ± 1.4
Reference	This study	Wolf et al., 2016

*In vivo and in silico data for specific growth rate μ [h^–1^], maximum substrate uptake rate qS_*max*_ [mmol⋅g^–1^⋅h^–1^], maximum cell dry weight [g⋅l^–1^] and integration of carbon into biomass Y_*C*_ [%] of S. solfataricus P2 after growth on phenol and D-glucose. In vivo values represent the average of three independent experiments. Errors represent the standard deviation between the experiments.*

To improve our metabolic model of *S. solfataricus* P2 for modeling phenol metabolism, we further analyzed the macromolecular composition of the organism after growth on phenol (Supplementary Material S3), as it has been shown that biomass composition could strongly depend on the environmental conditions (Neidhardt, 1963). Phenol grown cells show a similar composition of the nucleic acid fractions as compared to cells grown on D-glucose (RNA: 5.4 ± 0.5%, DNA: 1.1 ± 0.1%; Wolf et al., 2016). However, protein content of phenol grown cells is strongly decreased to only 47 ± 2.3% (D-glucose protein content: 64.3 ± 3.4%).

To investigate if the secretion of carbon containing compounds contributes to the poor conversion of carbon into biomass, we analyzed supernatants of *S. solfataricus* P2 after growth on phenol by GC-MS. Only small amounts of hydroquinone and oxalate were identified in the supernatants during the exponential growth phase (Supplementary Material S4). The oxalate amount in the media increases slightly in the stationary phase, whereas the hydroquinone concentration decreases. In contrast, none of these compounds could be detected under glucose growth conditions.

Estimating the conversion of carbon into biomass during phenol degradation using the metabolic model, results in an incorporation value of 14.3% (Table 1), which is consistent with the experimental findings. The model predicted twice as much oxygen consumption in simulations with phenol (15.0 mmol/g_*CDW*_/h) compared to glucose (7.2 mmol/g_*CDW*_/h). The respiratory activity increased in a similar manner.

### The Metabolic Response of *S. solfataricus* P2 to Phenol

**FIGURE 1:**
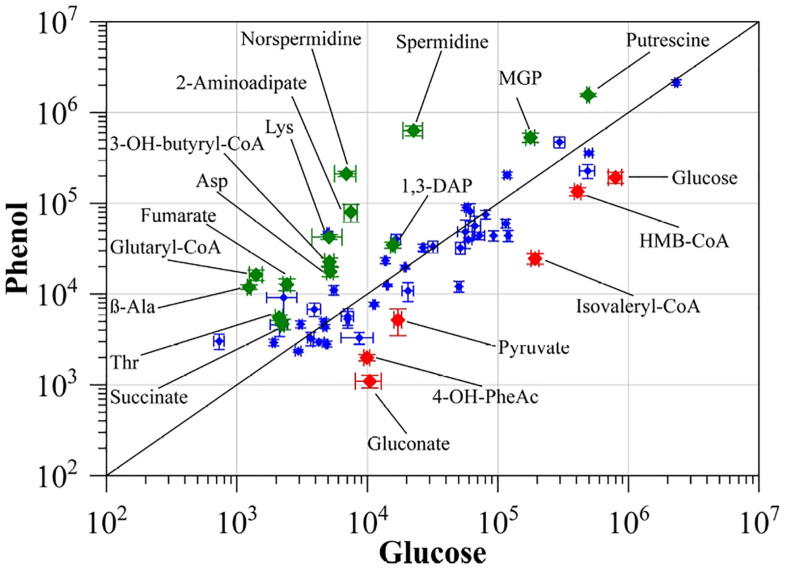
Comparison of intracellularly detected metabolites of *S. solfataricus* P2 grown on either phenol or D-glucose as sole carbon source. Normalized peak areas were plotted on a double logarithmic scale. Green diamonds: more abundant in cells grown on phenol, red diamonds: more abundant in cells grown on D-glucose. Only identified metabolites with significantly altered concentrations (*p* < 0.01) and at least twofold differences between both conditions were labeled. Values represent the average of three independent cultivations. Errors represent the standard error between the experiments. 1,3-DAP, 1,3-diaminopropane; MGP, methyl-α-D-glucopyranoside; HMB-CoA, 3-hydroxy-2-methylbutanoyl-CoA; 3-OH-butanoyl-CoA, 3-hydroxybutanoyl-CoA; 4-OH-PheAc, 4-hydroxy-phenylacetate.

To monitor changes in the metabolic activity of *S. solfataricus* P2 after growth on phenol as sole carbon source, we compared the intracellular metabolite proportions in the late exponential growth phase to cells grown on D-glucose as reference (Figure 1, Table 2, and Supplementary Material S5).

**TABLE 2:** Overview of detected metabolites of *S. solfataricus* P2 after growth on phenol.

Phenol metabolomics	
Metabolites (total)	77
CoA derivatives	17
Identified metabolites	40
Unidentified metabolites	20
Regulated compounds*	39
With *p*-value < 0.01	24
Detected only during growth on phenol	15

*Compounds with at least twofold change and p-value < 0.01 or not detectable under D-glucose reference condition.

**FIGURE 2:**
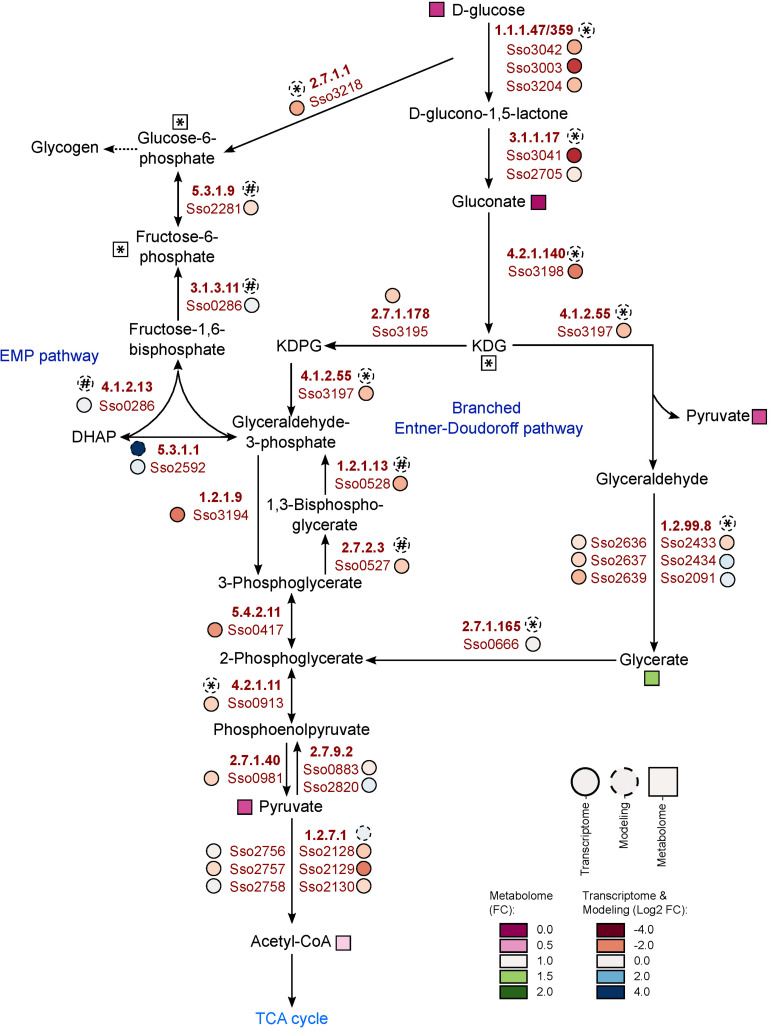
Metabolic and transcriptomic changes within the Entner-Doudoroff pathway and the gluconeogenesis during growth of *S. solfataricus* P2 on phenol Relative metabolite concentrations (squares) were expressed as fold-changes compared to the reference D-glucose. Transcript abundance (solid circles) and flux distributions (dashed circles) were expressed as Log2 fold-changes compared to the reference D-glucose. Asterisks: Data are only available under glucose conditions. Hash: Data are only available under phenol conditions. Dotted arrows indicate multiple enzymatic reactions. KDG, 2-keto-3-deoxygluconate. This pathway is available at MetaboMAPS (accession: P101).

The most obvious metabolic changes after growth on phenol were observed for the components of the glycolytic Entner-Doudoroff pathway and gluconeogenesis (Figure 2 and Table 3). We observed strongly decreased amounts not only for glucose, but also for gluconate, pyruvate and 2-keto-3-deoxygluconate, with the latter being only detected under glucose reference conditions (Table 3). Furthermore, the phosphorylated sugars, fructose-6-phosphate and glucose-6-phosphate, which belong to the gluconeogenic Embden-Meyerhof-Parnas pathway, as well as several other sugars (e.g., ribulose, xylulose, fructose) were also detected only with glucose as sole carbon source (Figure 2 and Table 3).

**TABLE 3:** Phenol or D-glucose specific metabolites.

Phenol	D-glucose
Acetoacetyl-CoA*	Erythritol
Adenine	Erythronic acid
Butanoyl-CoA	Fructose
Catechol	Fructose-6-phosphate
Glucosamine	Glucose-6-phosphate
Guanine	Glycerol-3-phosphate
Malonyl-CoA	Homoserine
	KDG
	Lactate
	Isomaltulose
	Proline
	Ribulose
	Xylulose

Identified metabolites that were only detected in cells of S. solfataricus P2 grown on either phenol or D-glucose as sole carbon source. KDG, 2-keto-3-deoxygluconate. *Compound is normally not stable during the extraction process, thus detection accounts for high intracellular concentration.

**FIGURE 3:**
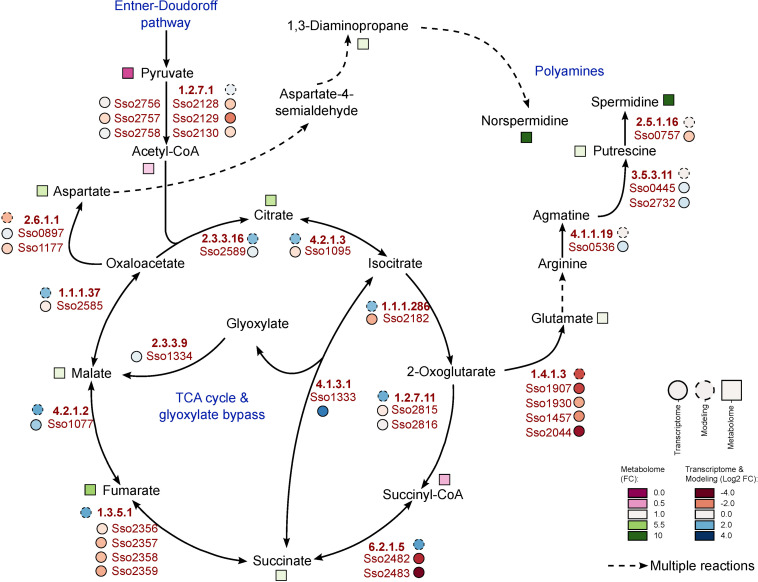
Metabolic changes within the TCA cycle during growth of *S. solfataricus* P2 on phenol. Relative metabolite concentrations (squares) were expressed as fold-changes compared to the reference D-glucose. transcript abundance (solid circles) and flux distributions (dashed circles) were expressed as Log2 fold-changes compared to the reference D-glucose. Dotted arrows indicate multiple enzymatic reactions. Note that the fold-changes of spermidine and norspermidine exceeds the color scale with values of 28 and 30, respectively. This pathway is available at MetaboMAPS (accession: P103).

As degradation of phenol finally leads to formation of pyruvate and acetyl-CoA (Shingler et al., 1992; Sridevi et al., 2012), the TCA cycle represents the entry point to central carbon metabolism for this carbon source. From the metabolites of the TCA cycle we observed significantly increased amounts only for succinate (2.09-fold) and fumarate (5.27-fold). Malate and citrate show increased concentrations by more than twofold with respect to the glucose reference, but these changes were not statistically significant (Figure 3).

In addition to the changes in the central carbon metabolism, we observed noticeable changes in polyamine formation if *S. solfataricus* P2 is cultivated with phenol as sole carbon source. Under these conditions, the relative concentrations of spermidine and norspermidine were increased by approximately 28- and 31-fold, as compared to the reference condition. Furthermore, elevated, but less pronounced levels were also found for 1,3-diaminopropane and putrescine (Figure 3).

In addition to small polar metabolites, we also monitored changes in the coenzyme A content of the cells between the different growth conditions. If *S. solfataricus* P2 grows on phenol as carbon source, we found a strong accumulation of glutaryl-CoA (11.57-fold) and 3-hydroxy-butanoyl-CoA (4.4-fold). Furthermore, butanoyl-CoA, malonyl-CoA and acetoacetyl-CoA could only be detected under phenol growth conditions (Table 3). It should be noted, that acetoacetyl-CoA is normally not stable during the extraction process, thus the detection of this compound in phenol cell extracts accounts for a high intracellular concentration. In conjunction with an increased coenzyme A biosynthesis, we also found the precursor beta-alanine (Genschel, 2004; Tomita et al., 2014) accumulating during growth of *S. solfataricus* P2 on phenol (Figure 1).

Finally, decreased concentrations were found for 3-hydroxy-2-methylbutanoyl-CoA (0.33-fold) and isovaleryl-CoA (0.13-fold) (Figure 1), which are both connected to amino acid degradation in *S. solfataricus* P2 (Stark et al., 2017).

### Global Transcriptomic Changes of *S. solfataricus* P2 During Growth on Phenol

Amongst others, phenol is formed during lignin degradation and toxic to most organisms. Its utilization as a carbon source is less common and requires a complex metabolic adaption. To investigate the cellular changes of this adaption process, we analyzed the global transcriptomic response of *S. solfataricus* P2 to growth on phenol, using RNASeq analysis and growth on D-glucose as reference condition. From the 3,213 genes encoded on the *S. solfataricus* P2 genome, we could detect 2,760 genes to be actively transcribed under both growth conditions. Out of these, 274 genes showed significantly altered abundance with changes of at least fourfold (log2FC > 2, *p* <0.01) (Table 4 and Supplementary Material S6).

**TABLE 4:** Overview of differential expressed protein classes in *S. solfataricus* P2 after growth on phenol.

Class	Up-regulated	Down-regulated
Total number of regulated genes	100	174
Growth rate related genes*	3	47
Nucleotide metabolism	0	8
Amino acid metabolism	0	18
Membrane proteins**	18	11
Transposable elements	12	3
Uncharacterized proteins	40	24

*Includes proteins involved in transcription and translation processes. **Proteins with at least two transmembrane domains.

The majority of the 100 up-regulated genes found under phenol growth conditions belong either to membrane proteins, mobile elements or uncharacterized proteins (Table 4), In contrast, the majority of the 174 down-regulated transcripts are associated with growth rate related genes, including ribosomal proteins, tRNA synthetases and enzymes that are involved in replication, translation and transcription processes (Table 4). Further a strong down regulation of genes belonging to amino acid and nucleotide biosynthesis was observed.

Among the strongly induced transcripts observed under phenol growth conditions, we found four of the seven subunits of a monooxygenase gene cluster (Sso1225–Sso1233) showing significant differential expression with fold-changes ranging from 59 up to 384-fold (Table 5). The remaining three subunits of these gene cluster also show high transcription levels compared to growth on D-glucose (fold-change 10 up to 73-fold), but due to higher standard errors, these changes were not statistically significant (Table 5). Interestingly, together with the catechol-2,3-dioxygenase (Sso1223), this gene cluster has already been shown to be involved in phenol degradation in *S. solfataricus* (Izzo et al., 2005). Furthermore, several genes located upstream of the monooxygenase gene cluster also show strong induction under phenol growth conditions, including an alcohol dehydrogenase (Sso1220), two acetoacetate decarboxylase related enzymes (Sso1221 and Sso1222) and methylmalonate-semialdehyde dehydrogenase (Sso1218).

**TABLE 5:** Overview of the most differentially expressed genes in *S. solfataricus* P2 cells after growth on phenol.

Locus tag	EC class	Product	Fold-change^*a*^	*p*-value^*b*^
Sso1228	1.14.13.244	Toluene-4-monooxygenase system protein A, carboxy end fragment (tmoA)	384 ± 160	6.02E-06
Sso1218	1.2.1.27	Methylmalonate-semialdehyde dehydrogenase (acylating)	382 ± 45	2.35E-97
Sso1220	1.1.1.1	Zn-dependent alcohol dehydrogenase	346 ± 128	2.51E-07
Sso1229		Toluene 4-monooxygenase, protein B (tmoB)	163 ± 64	1.57E-05
Sso2941	4.2.1.80	2-Oxopent-4-enoate hydratase	88 ± 21	2.54E-23
Sso1230	1.14.13.244	Toluene-4-monooxygenase system protein C (tmoC)	82 ± 33	1.06E-04
Sso1222	4.1.1.4	Acetoacetate decarboxylase related enzyme	82 ± 16	3.63E-17
Sso1221	4.1.1.4	Acetoacetate decarboxylase related enzyme	77 ± 19	2.37E-10
Sso1227	1.14.13.244	Toluene-4-monooxygenase system protein A, amino end fragment (tmoA)	73 ± 43	1.53E-02
Sso1233	1.14.13.244	Toluene-4-monooxygenase system protein E (tmoE)	59 ± 25	3.06E-04
Sso2690		CBS domain containing uncharacterized protein	58 ± 23	2.34E-05
Sso12083		Uncharacterized membrane protein	52 ± 21	1.13E-10
Sso1234		Predicted metal-sulfur cluster biosynthetic enzyme	45 ± 18	3.48E-04
Sso1129	1.8.98.1	Heterodisulfite reductase. subunit B	38 ± 19	9.41E-03
Sso1366		Transposase ISC1316	36 ± 7.2	3.23E-33
Sso1133	1.8.98.1	Predicted heterodisulfide reductase subunit	34 ± 16	4.58E-03
Sso1120	1.8.99.B1	Protein disulfide oxidoreductase	32 ± 7.2	9.57E-12
Sso1223	1.13.11.2	Catechol 2.3-dioxygenase	31 ± 20	5.90E-02
Sso1131	1.8.98.1	Heterodisulfide reductase. subunit A	26 ± 12	9.19E-03
Sso1125		DsrEFH-like family protein	26 ± 8.1	3.49E-05
Sso1504		Transposase ISC1316	25 ± 3.1	1.50E-37
Sso1127	1.8.98.1	Heterodisulfide reductase. subunit C	21 ± 5.7	8.11E-06
Sso1123	1.8.1.4	Dihydrolipoyl dehydrogenase	19 ± 6.3	1.72E-04
Sso1135	1.8.98.1	Heterodisulfide reductase. subunit B	19 ± 7.9	7.12E-03
Sso1231	1.14.13.244	Toluene-4-monooxygenase system protein D (tmoD)	18 ± 8.6	1.77E-02
Sso2785		Transposase ISC1316	15 ± 2.0	4.03E-23
Sso1134	1.8.98.1	Heterodisulfide reductase, subunit C	14 ± 6.7	2.65E-02
Sso1225	1.14.12.-	Toluene 1,2-dioxygenase system ferredoxin-NAD (+) reductase component (todA)	9.7 ± 6.5	1.96E-01
Sso2178	1.2.1.11	Malonyl-CoA reductase	9.1 ± 2.0	5.37E-06
Sso1333	4.1.3.1	Isocitrate lyase	7.2 ± 0.3	3.94E-18
Sso0120		Assembly ATPase involved in the assembly of the UV induced pili	7.0 ± 0.8	6.10E-17
Sso2942	1.2.99.8	Glyceraldehyde dehydrogenase (FAD-containing), large chain	6.7 ± 0.6	8.43E-17
Sso3203	6.2.1.40	4-hydroxybutyrate-CoA ligase	6.1 ± 1.6	9.00E-04
Sso0117		Pilin subunit of UV induced pili	5.7 ± 0.9	3.01E-13
Sso10828	1.10.3.13	Caldariellaquinol oxidase (SoxABC), cytochrome B subunit, C-terminal part (soxC)	5.5 ± 1.4	4.18E-04
Sso2537	4.1.1.32	Phosphoenolpyruvate carboxykinase (GTP)	4.7 ± 0.4	1.19E-11
Sso2657	1.10.3.13	Caldariellaquinol oxidase (SoxABC), cytochrome aa3 subunit (soxB)	4.5 ± 1.1	1.18E-03
Sso7986		Transposase ISC1439	4.1 ± 0.8	2.02E-06
Sso2773	4.2.1.80	2-Oxopent-4-enoate hydratase	2.1 ± 0.4	9.07E-03
Sso0700		50S ribosomal protein L19e	0.03 ± 0.01	2.73E-32
Sso0594	5.3.1.16	1-(5-phospho ribosyl)-5-[(5-phosphoribosylamino) carboxamide isomerase (hisA)	0.04 ± 0.004	1.39E-34
Sso0068		30S ribosomal protein S9	0.05 ± 0.01	6.29E-32
Sso2972		Caldariellaquinol oxidase (H+-transporting), sulfocyanin (soxE)	0.05 ± 0.03	6.02E-06
Sso10704		Uncharacterized protein	0.05 ± 0.02	2.35E-97
Sso0712		30S ribosomal protein S3	0.05 ± 0.01	1.52E-29
Sso5140	2.7.7.6	DNA-directed RNA polymerase subunit N	0.05 ± 0.01	2.30E-29
Sso0704		50S ribosomal protein L5	0.06 ± 0.01	2.09E-28
Sso0069		50S ribosomal protein L13	0.06 ± 0.01	2.09E-28
Sso2483	6.2.1.5	Succinyl-CoA synthetase, beta subunit (sucC)	0.07 ± 0.02	1.88E-23
Sso2970	1.10.3.13	Caldariellaquinol oxidase (H+-transporting), cytochrome b (soxG)	0.09 ± 0.05	1.41E-24
Sso2971		Caldariellaquinol oxidase (H+-transporting), Rieske Fe-S protein (soxF)	0.09 ± 0.05	1.43E-24
Sso2968	1.10.3.13	Caldariellaquinol oxidase (H+-transporting) (soxI)	0.10 ± 0.06	1.05E-21
Sso1915		Transposase ISC1913	0.11 ± 0.03	1.30E-16
Sso2482	6.2.1.5	Succinyl-CoA synthetase, alpha subunit (sucD)	0.12 ± 0.02	2.21E-17
Sso3003	1.1.1.47	Glucose-1-dehydrogenase	0.14 ± 0.02	4.15E-15
Sso1830		Transposase ISC1778	0.18 ± 0.02	2.32E-12
Sso3194	1.2.1.9	Non-phosphorylating NAD(P)-dependent glyceraldehyde 3-phosphate dehydrogenase	0.23 ± 0.06	1.99E-10
Sso3198	4.2.1.140	Gluconate/galactonate dehydratase	0.24 ± 0.01	2.59E-09
Sso2182	1.1.1.42	Isocitrate dehydrogenase (NADP^+^)	0.39 ± 0.04	7.94E-05
Sso3197	4.1.2.20/4.1.2.21	Bifunctional 2-keto-3-deoxy-(6-phospho)gluconate/galactonate aldolase	0.44 ± 0.01	6.58E-04

*Genes which are not significantly regulated but discussed in the text were also included. The fold-change represents the relative transcript abundance averaged from three independent experiments. S. solfataricus P2 cells grown on D-glucose served as reference. Gene products were assigned using EnzymeDetector (Quester and Schomburg, 2011) or arCOG based annotations (Esser et al., 2011). ^*a*^Fold-change in relative abundance as compared to the D-glucose reference condition. ^*b*^P < 0.01 indicates statistically significant differential gene expression.*

Oxygen availability is a crucial factor for phenol metabolism (Comte et al., 2013), as it is the co-substrate of the monooxygenase, catalyzing the first step in phenol degradation. Interestingly, we found differential regulation in the expression of two different quinol oxidase complexes in *S. solfataricus* P2. Whereas the quinol oxidase complex SoxABC (Sso2656–Sso2662 and Sso10828) was significantly induced under phenol growth conditions (fold-changes: 3.1–7.1), the second quinol oxidase complex SoxM (Sso2968–Sso2973) showed a strong decrease in relative abundance, compared to the D-glucose reference condition (Table 5 and Supplementary Material S6). In addition, transcription of genes involved in the glycolytic Entner-Doudoroff pathway like the glucose-1-dehydrongease (Sso3003) or the GAP-dehydrogenase (Sso3194; Table 5) was significantly decreased.

We further found a gene cluster comprising the loci Sso1120–Sso1135 (fold-change 12–34) among the upregulated transcripts. Next to some proteins with unknown function, this gene cluster encodes for a putative heterodisulfide reductase (Sso1127 -Sso1135), a protein-disulfide oxidoreductase (Sso1120; Limauro et al., 2014) and a putative dihydrolipoyl dehydrogenase (Sso1123).

Furthermore, the data indicates a high plasticity of the genome, if *S. solfataricus* P2 is grown on phenol as carbon source. In total twenty-two transposable elements showed increased transcription under these conditions (Tables 4, 5 and Supplementary Material S6), although only 12 show expression levels above the median RPKM of the complete dataset. In contrast, during growth on D-glucose only three mobile elements show significantly increased expression (Table 4 and Supplementary Material S6). Finally, we found an increased transcription of the UV-inducible pili operon (Sso0117- Sso0121; fold-change 2.8–7.0) during growth on phenol, although two of the five subunits still show low relative expression levels (Supplementary Material S6).

## Discussion

### Global Response of *Saccharolobus solfataricus* P2 to Phenol and Metabolic Modeling

Compared to growth on glucose, phenol represents a poor source of carbon and energy for *Saccharolobus solfataricus* P2, as growth rate and biomass production were strongly decreased on this substrate (Table 1). A similar growth rate for batch cultures of *S. solfataricus* grown on phenol has been reported previously (Izzo et al., 2005). Furthermore, it has been shown from bioreactor cultivations that the growth rate of the organism on phenol is strongly dependent on the concentration of dissolved oxygen and decreases with reduced oxygen supply (Comte et al., 2013). Considering the fact that oxygen is the co-substrate of the mono- and dioxygenase catalyzing the first steps in phenol breakdown, it is evident that a sufficient oxygen supply is a crucial factor for efficient phenol degradation.

The strongly reduced growth rate during growth with phenol as carbon source is also reflected in the transcription of several growth rate related genes, as we found strongest down regulation of different ribosomal genes, tRNAs synthetases and genes involved in purine and amino acid metabolism (Tables 4, 5 and Supplementary Material S6). Interestingly, nearly all down regulated genes belonging to purine biosynthesis are involved in ATP consuming reactions. This has also been reported for *Bacillus subtilis* and *P. putida* during phenol exposure (Santos et al., 2004; Tam et al., 2006), suggesting that growth rate reduction prevents the organisms of consuming ATP for nucleotide biosynthesis and cell division and rather channeling the available energy to processes crucial for phenol adaptation.

Next to the reduced growth rate, we also observed a less efficient conversion of carbon into biomass if *S. solfataricus* is grown on phenol (13.8 ± 3.1%, Table 1). The carbon distribution is in concordance with that reported from Christen et al. (2011), if glucose adapted cells were grown with phenol as sole carbon source, indicating a higher respiratory activity on this substrate.

In conjunction with an increased oxygen demand, we found that phenol influences the expression of different quinol oxidase complexes in *S. solfataricus* as the SoxABC complex is induced, whereas a second quinol oxidase complex (SoxM) showed a strongly decreased abundance (Table 5 and Supplementary Material S6). A similar expression pattern has been previously reported when *S. solfataricus* is exposed to low oxygen concentrations (Simon et al., 2009). This indicates a higher oxygen affinity of the SoxABC complex, which is linked to a smaller number of protons pumped via the membrane per electron transferred (Gleißner et al., 1997; Komorowski et al., 2002). Thus, increased expression of this complex under phenol growth conditions emphasizes the importance of sufficient oxygen supply for efficient phenol utilization.

The metabolic model of *S. solfataricus* considers transcriptomic and metabolic changes that are determined in this study, meaning it takes the different quinol oxidases into account. The model is in consent with *in vivo* studies by showing a higher oxygen consumption in simulations with phenol. 16.5% of the oxygen is already used for the oxidation of phenol to catechol. The model showed also a higher respiratory activity leading to ∼40% more oxygen consumption than in glucose simulations and a lower incorporation of carbon into the biomass. The low carbon yield and growth rate on phenol results from a higher demand of oxygen which leads to the usage of a less efficient terminal oxidase and further decreases the ATP yield per substrate.

### Reconstruction of Phenol Degradation in *S. solfataricus* P2

Previous studies already predicted the phenol *meta-*cleavage pathway to be active in *S. solfataricus* growing on phenol (Izzo et al., 2005; Comte et al., 2013). On the metabolic level clear evidence for the activity of this pathway suffers from the missing commercial availability of intermediates that could serve as standards for mass spectra libraries. Only catechol, *cis, cis-*muconate and 3-oxoadipate are available, the latter two compounds representing intermediates of the *ortho-*cleavage pathway (Ornston, 1966; Feist and Hegeman, 1969). With the exception of catechol none of these intermediates could be detected in our experiments, indicating a minor importance of the *ortho-*route in this thermophilic archaeon.

**FIGURE 4:**
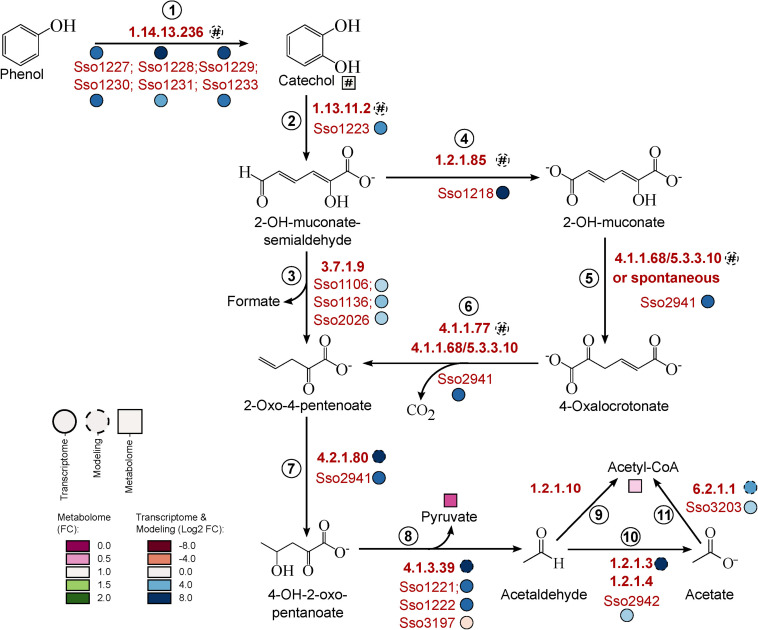
Refinement of phenol *meta-*degradation pathway in *S. solfataricus* P2. For simplicity, additional Co-substrates and Co-factors were not shown. Proteins predicted to be involved in phenol degradation were indicated by the corresponding gene loci. Relative metabolite concentrations (squares) were expressed as fold-changes compared to the reference D-glucose. Transcript abundance (solid circles) and flux distributions (dashed circles) were expressed as Log2 fold-changes compared to the reference D-glucose. Hash: Data are only available under phenol conditions. 2-OH-muconate-semialdehyde, 2-hydroxymuconate-semialdehyde; 2-OH-muconate, 2-hydroxymuconate; 4-OH-2-oxo-pentanoate, 4-hydroxy-2-oxo-pentanoate. This pathway is available at MetaboMAPS (accession: P100).

Independent of which route for phenol breakdown is used, the initial step is the transformation to catechol, which requires the presence of an active monooxygenase. A putative monooxygenase gene cluster (Sso1225–Sso1233) involved in this reaction has already been identified by Izzo et al. (2005). Accordingly, we found that the expression of all genes of this cluster was strongly induced under phenol growth conditions (Figure 4, reaction 1).

The following transformation of catechol to 2-hydroxymuconate-semialdehyde requires a ring opening reaction by catechol-2,3-dioxygenase. The corresponding homolog in *S. solfataricus* (Sso1223) has also been characterized before (Izzo et al., 2005; Chae et al., 2007) and is located in the same gene cluster as the monooxygenase. Concordantly, we observed an increased expression of this gene under phenol growth conditions (Figure 4, reaction 2, Table 5). In addition to the characterized protein a second homolog of the dioxygenase exists on the *S. solfataricus* genome (Sso2054, Izzo et al., 2005), but this gene showed very low expression levels under both, phenol and glucose growth conditions (Supplementary Material S6), suggesting no specific importance of this protein for phenol degradation.

2-Hydroxymuconate-semialdehyde is then degraded to 2-oxo-4-pentenoate, which could be achieved by two different metabolic routes (Figure 4, reactions 3–6). For none of these reactions the participating enzymes in *S. solfataricus* are known, because no homologous to known bacterial or eukaryotic enzymes could be identified in the genome. The direct conversion requires hydrolase activity (Figure 4, reaction 3). We found three putative hydrolases to be induced under phenol growth conditions compared to the glucose reference (Sso1106, Sso1136, Sso2026). However, the expression level for these genes were very low under both growth conditions (Supplementary Material S6), indicating a minor importance of the hydrolase cleavage of 2-hydroxymuconate-semialdeyhde. An alternative pathway for conversion of the semialdehyde involves the oxidation to 2-hydroxymuconate (Figure 4, reaction 4). Previously, Comte et al. (2013) reported the formation of small amounts of 2-hydroxymuconate and 4-oxalocrotonate in batch cultures of *S. solfataricus*, providing evidence for the activity of this branch. The genome encodes for five members of the aldehyde dehydrogenase superfamily: Two of them, Sso3117 and Sso3194, have been extensively studied and proven to be involved in different sugar degradation pathways (Brouns et al., 2006; Ettema et al., 2008). The remaining candidates (Sso1218, Sso1629, and Sso1842) show approximately 30% identity to characterized 2-hydroxymuconate-semialdehyde dehydrogenases of *Pseudomonas* sp. (Inoue et al., 1995). For Sso1629 and the previously characterized succinate-semialdehyde dehydrogenase (Sso1842) (Esser et al., 2013) no change in transcript abundance was observed in response to carbon source (Supplementary Material S6).

The most promising candidate is Sso1218 based on transcriptomic data, which showed a 382-fold increase in expression in cells grown on phenol (Table 5). Moreover, this gene is located in the same cluster as the mono- and dioxygenase. Interestingly, Sso1218 has previously been characterized as methylmalonate-semialdehyde dehydrogenase and is supposed to be strictly coenzyme A-dependent and involved in degradation of branched-chain amino acids. However, the enzyme showed an unusual low activity for its physiological substrate (Esser et al., 2013). Recently, it has been shown that branched-chain amino acids were not primary degraded via the classical routes in *S. solfataricus*. In the same study Sso1218 did not show differential expression during growth on amino acids (Stark et al., 2017). This indicates that not methylmalonate-semialdehyde but rather 2-hydroxymuconate-semialdehyde is the physiological substrate of this enzyme.

In the next step of the phenol *meta-*cleavage pathway, 2-hydroxymucoante is isomerised to 4-oxalocrotonate (Figure 4, reaction 5). This reaction has been shown to occur spontaneously in cell extracts of *Azotobacter* sp. and *Comamonas* sp. (Sala-trepat and Evans, 1971; Liu et al., 2007), so it is likely that this might also be true for *S. solfataricus.* However, an involvement of the strongly induced protein Sso2941 in the isomerisation reaction cannot be excluded (fold-change 88 ± 21, Table 5). This gene is currently annotated as 2-oxo-4-pentanoate hydratase and would therefore be involved in the formation of 4-hydroxy-2-oxo-pentanoate (Figure 4, reaction 7). However, Sso2941 also shows similarity to a bifunctional isomerase/decarboxylase from *Escherichia coli* (32% identity, *e*-value: 8e-22) which catalyses the isomerisation of 5-carboxymethyl-2-hydroxymuconate as well as the subsequent decarboxylation to 2-oxo-hetp-4-enedioate (Jervis et al., 1996). In *E. coli* these reactions are part of the 4-hydroxyphenylacetate meta-degradation pathway, which is closely related to the catechol pathway. Furthermore, Sso2941 belongs to the fumarylacetoacetate hydrolase family, which is also responsible for the decarboxylation of 4-oxalocrotonate (Figure 4, reaction 6). Thus, suggesting that Sso2941 may be involved in different steps of the phenol *meta-*degradation pathway.

Finally, the cleavage of 4-hydroxy-2-oxo-pentanoate into pyruvate and acetaldehyde requires the action of an aldolase (Figure 4, reaction 8). The 2-keto-3-deoxy-(6-phospho)gluconate/galactonate aldolase (Sso3197) of *S. solfataricus* has been already shown to possess a broad substrate specificity (Ahmed et al., 2005; Nunn et al., 2010), and thus might be able to catalyze the cleavage of 4-hydroxy-2-oxo-pentanoate. Additionally, we observed a strong induction of two proteins of yet unknown function (Sso1221 and Sso1222, Table 5). Both proteins contain conserved domains assigning them to the acetoacetate decarboxylase superfamily. This superfamily also includes the enturacidine biosynthesis protein MppR, which acts as an aldolase, catalyzing the condensation of pyruvate and 4-imidazolecarboxaldehyde (Burroughs et al., 2013). Considering the reversible nature of aldolase reactions, we analyzed the sequences of Sso1221 and Sso1222 for their similarity to known MppR proteins and found an identity of 21–29% (*E*-values: 2e^–14^–6e^–34^) to the bacterial enzymes. In view of the structural diversity, archaeal proteins often differ strongly from their bacterial counterparts, thus we suggest a possible involvement of the two acetoacetate decarboxylase-related enzymes in phenol degradation. This was supported further by structural modeling using Swiss-Model: the characterized MppR protein from *Streptomyces hygroscopicus* [PDB:4JM3; Burroughs et al. (2013)] was selected as model template with 94% coverage and a GMQE (Global Model Quality Estimation score) of 0.64 for Sso1221 and 86% coverage and a GMQE of 0.57 for Sso1222. Both proteins showed high structural conservation especially in the center of the homo-tetramer (Supplementary Material S7).

In the last step of phenol degradation acetaldehyde has to be converted to acetyl-CoA. Based on our data, this includes the conversion of acetaldehyde to acetate with subsequent formation of acetyl-CoA (Figure 4, reactions 10 and 11). This mechanism is already proposed to be active in *S. solfataricus* during growth on ethanol (Chong et al., 2007). To date no acetaldehyde dehydrogenase is known in *S. solfataricus*, but we observed induction of a currently annotated glyceraldehyde dehydrogenase (Sso2942). The enzyme shares 34% identity to the glyceraldehyde oxidoreductase of *S. tokodaii* which has shown to be active on acetaldehyde (Wakagi et al., 2016). Together with the also induced 4-hydroxybutyrate-CoA ligase (Sso3203, Table 5) it appears likely, that acetaldehyde is first oxidized to acetate, before it is channeled into the TCA cycle via formation of acetyl-CoA. Finally, excess acetaldehyde could probably also be reduced by the action of an alcohol dehydrogenase, as we found a member of this enzyme amongst the most upregulated transcripts (Sso1220, fold-change 346 ± 128 Table 5). A similar up-regulation of an alcohol dehydrogenase has been previously reported for *Pseudomonas* sp. during growth on phenol (Collinsworth et al., 1973; Shingler et al., 1992). Produced ethanol would partially evaporate at growth temperatures of 80 °C. In addition to higher CO_2_ production levels, this may also contribute to the very low integration of carbon into the biomass we observed for growth of *S. solfataricus* on phenol (Table 1).

### Phenol Induces Multiple Stress Responses in *S. solfataricus* P2

It is well known that benzene and its derivatives cause an increased formation of reactive oxygen species in pro- and eukaryotic cells, which could lead to oxidative damage of the DNA (Winn, 2003; Santos et al., 2004). Microorganisms which are able to use phenol as carbon source possess defense mechanisms against oxidative stress, often including the up-regulation of antioxidant proteins (Santos et al., 2004). In *S. solfataricus* growth on phenol induces the transcription of a gene cluster encoding for a putative heterodisulfide reductase, as well as for a protein-disulfide oxidoreductase and a putative dihydrolipoyl dehydrogenase (Table 5). The protein-disulfide oxidoreductase (Sso1120) has been characterized previously and does not to interact with peroxiredoxins from *S. solfataricus* (Limauro et al., 2014). However, this protein interacts with a putative dihydrolipoyl dehydrogenase (Sso1123), which has been shown to take part in an alternative antioxidant system in *Mycobacterium tuberculosis* (Koshkin et al., 2004). Thus it is suggested that these proteins, together with the heterodisulfide reductase may represent a system against oxidative stress in *S. solfataricus* in presence of elevated phenol concentrations.

In addition to antioxidant proteins, polyamines have been shown to function as radical scavengers, protecting cells from oxidative stress (Ha et al., 1998; Jung et al., 2003). We found a strong accumulation of different polyamines during growth on phenol, which was absent under the glucose reference condition (Figure 3). Thus, our results indicate that *S. solfataricus* P2 uses both, an antioxidant system and the accumulation of polyamines to overcome the increased oxidative stress the organism is exposed to during growth on phenol.

When *S. solfataricus* P2 is grown on phenol, a high plasticity of the genome is observed, as we found 22 transposable elements upregulated between 4- and 34-fold (Table 5 and Supplementary Material S6). Mobile elements normally enable rearrangement of the genome by homologous recombination which leads to loss or induction of gene activity (Syvanen, 1994). In *S. solfataricus*, these mobile elements are further responsible for the relatively high spontaneous mutation rate (Martusewitsch et al., 2000). *S. solfataricus* contains several classes of predicted mobile elements, some of them have been shown to be mobile on the experimental level while others were shown to be non-mobile (Brügger et al., 2002; Filee et al., 2007). Among the upregulated transposable elements, we found 14 sequences belonging to the ISC1316 class, which has no known function so far and which was predicted to be non-mobile (Filee et al., 2007). However, we also found members of the ISC1439 class strongly induced under phenol growth conditions. For this class mobility causing mutations has already been reported and there is evidence of mobility for other ISC classes (Brügger et al., 2002). Such a rearrangement of the genome could serve as another mechanism to overcome phenol induced stresses and toxicity, probably by inducing expression of the required proteins. A similar increase in expression of transposable elements has previously been reported for *S. solfataricus* after exposure to heat shock (Tachdjian and Kelly, 2006). Furthermore, genome rearrangement may also mediate DNA exchange or repair. We found an increased transcription of the gene cluster encoding for the UV-induced pili system. The formation of these pili in *S. solfataricus* mediates cell-cell contact and formation of cell aggregates which would allow for DNA exchange between cells, thus enabling DNA repair by homologous recombination (Fröls et al., 2007).

## Conclusion

In this work we describe the global metabolic and transcriptional response of *Saccharolobus solfataricus* P2 during growth with phenol as sole carbon source. Our data underline the importance of the phenol *meta*-degradation pathway in *S. solfataricus*, as intermediates of the *ortho*-route were not detected. Based on our data, we provide enzyme candidates for the degradation of phenol by the *meta-*degradation pathway downstream of 2-hydroxymucconic acid. Furthermore, our data clearly showed that the already characterized enzymes of phenol degradation acting upstream of 2-hydroxymucconic acid are strongly up-regulated during growth on phenol, compared to the reference condition. Although *S. solfataricus* uses phenol for biomass production, it causes several stress reactions, including genome rearrangement, polyamine accumulation and expression of antioxidant proteins. A higher oxygen demand for substrate oxidation leads to the usage of a less efficient terminal oxidase and an increased respiratory activity to meet the energy demand of the cell. This further contributes to an even lower ATP yield per substrate and to the very poor incorporation of carbon into biomass which was furthermore confirmed by the metabolic model.

## Data Availability Statement

The datasets presented in this study can be found online at the FAIRDOMHub platform via: https://fairdomhub.org/investigations/401. The metabolic model is available at the MMTB platform via: https://mmtb.brenda-enzymes.org/models/view/3.

## Author Contributions

JW, JKa, BS, DS, and MN-S conceived the idea and designed the experiments. JW, AA, and MN-S performed the experiments. JKo performed the computational modeling. JW, JKo, AA, and MN-S analyzed the data. JW, JKo, and MN-S wrote the manuscript with input of all authors. All authors edited the manuscript and approved the final manuscript.

## Conflict of Interest

The authors declare that the research was conducted in the absence of any commercial or financial relationships that could be construed as a potential conflict of interest.

## Footnotes

1 https://mmtb.brenda-enzymes.org
