# Supplementary material for: Utilization of Phenol as Carbon Source by the Thermoacidophilic Archaeon Saccharolobus solfataricus P2 Is Limited by Oxygen Supply and the Cellular Stress Response
Source: Front Microbiol. 2021 Jan 8;11:587032. doi: 10.3389/fmicb.2020.587032 (PMC7820114; doi:10.3389/fmicb.2020.587032)

Supplementary Material

**Supplementary Material S1**: **Maximum optical densities of *S. solfataricus* P2 reached at different phenol concentrations.** *no growth detectable.


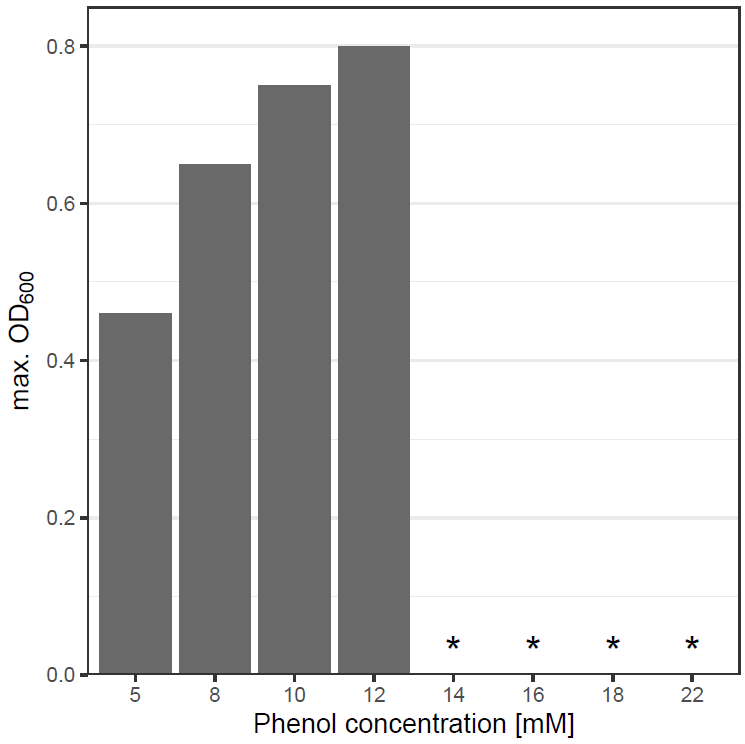


**Supplementary Material S2:** **Growth curves of *S. solfataricus* P2 at 10 mM phenol (red dots) and 22 mM D-glucose (black dots).** Growth on D-glucose has been published previously (Wolf *et al.*, 2016).


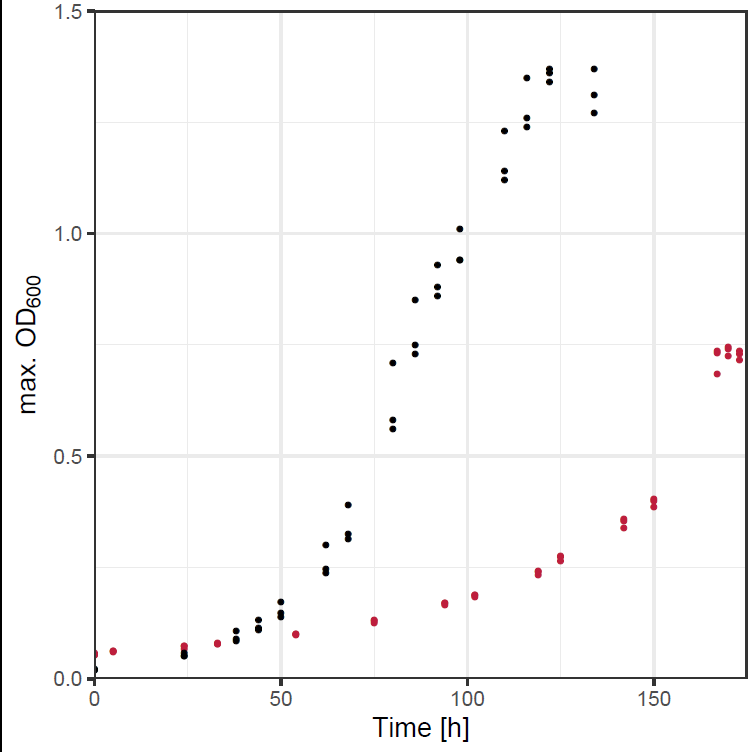


Supplementary Material S3: Biomass composition of *S. solfataricus* P2 after growth on phenol.

Composition of major biomass fractions of *S. solfataricus* P2 after growth on phenol as sole carbon source. Values represent the average of three independent experiments. Errors represent the standard deviation between the experiments.

| **Carbon source** | **Component** | **Cellular content [% (*w/w*)]** |
| --- | --- | --- |
| **Phenol** | Proteins | 47.0 ± 2.3 |
|  | RNA | 4.2 ± 0.04 |
|  | DNA | 1.2 ± 0.1 |
|  | Other | 48.0 |

Supplementary Material S4: Secretion of organic compounds by *S. solfataricus* P2 after growth on phenol as sole carbon source. Normalized peak areas are plotted against the corresponding time points. 142 h represents the exponential growth phase. whereas 167 h represents stationary growth. Blue: hydroquinone. red: oxalate. Values represent the average of three independent cultivations. Errors represent the standard error between the experiments.


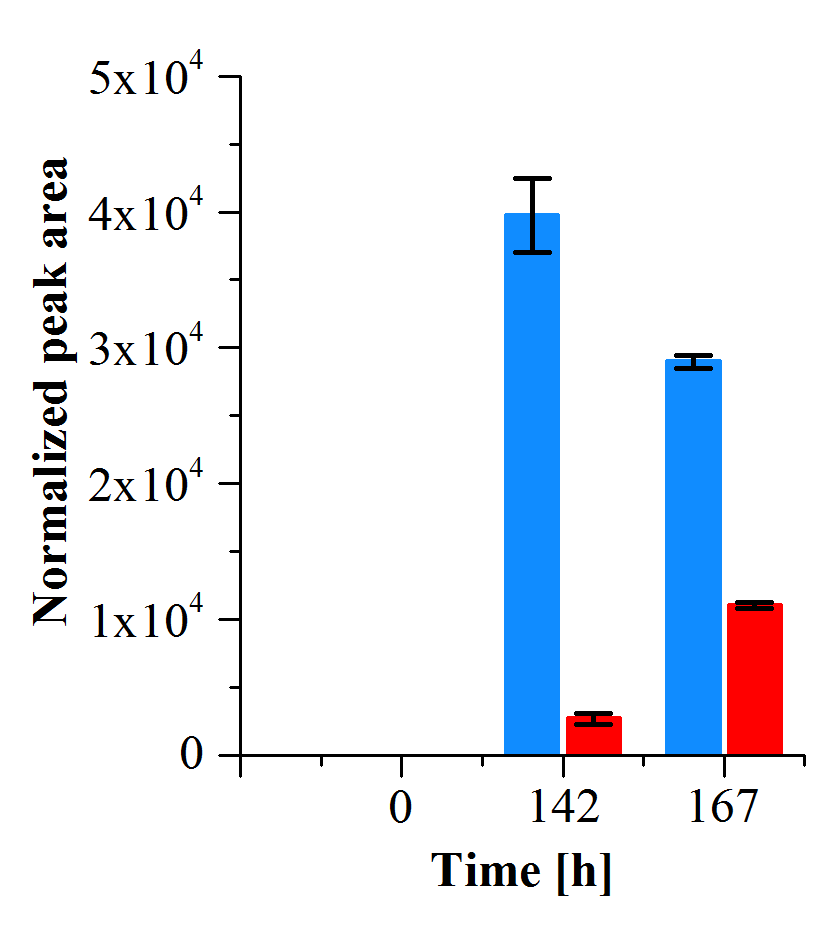


Supplementary Material S7: Modelling of Sso1221 and Sso1222 against the MppR protein of *Streptomyces hygroscopicus* (PDB:4JM3). Blue colored parts represent areas of high homology between the proteins. Red colored parts represent differences in protein structure.


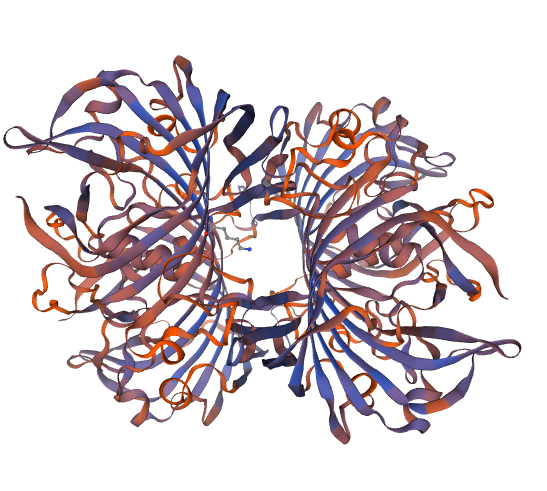

Supplement: Supplementary file 1 [file Table_1.DOCX]
